# Managers’ Compensation in a Mixed Ownership Industry: Evidence from Nursing Homes

**DOI:** 10.3389/fpubh.2016.00283

**Published:** 2016-12-26

**Authors:** Sean Shenghsiu Huang, Richard A. Hirth, Dean G. Smith

**Affiliations:** ^1^Department of Health Systems Administration, Georgetown University, Washington, DC, USA; ^2^Department of Health Management and Policy, University of Michigan, Ann Arbor, MI, USA; ^3^Louisiana Health Sciences Center, School of Public Health, New Orleans, LA, USA

**Keywords:** nursing homes, non-profit, ownership, compensation, quality, incentives

## Abstract

An extensive literature is devoted to differences between for-profit and non-profit health-care providers’ prices, utilization, and quality. Less is known about for-profit and non-profit managers’ compensation and its relationship with financial and quality performance. The aim of this study is to examine whether for-profit and non-profit nursing homes place differential weights on financial and quality performance in determining managers’ compensation. Using a unique 8-year dataset on Ohio nursing homes, fixed-effect regression models of managers’ compensation include financial and quality performance as well as other explanatory variables concerning firm and market characteristics and manager qualifications. Among for-profit nursing homes, compensation of owner-managers and non-owner managers are compared. Compensation of for-profit managers is significantly positively associated with profit margin and return-on-assets, while compensation of non-profit managers does not exhibit any consistent relationship with financial measures. Compensation of neither for-profit nor non-profit managers is significantly related to quality measures. Nursing home size and managers’ years of experience are the only consistent determinants of compensation. Owner-managers earn significantly higher compensation than non-owner managers and their compensation is less related to nursing home performance. Finding that home size and experience are strong determinants of compensation, and the association with ownership and financial performance for for-profit nursing homes are as expected. The insignificant relationship between compensation and quality performance is potentially troublesome.

## Introduction

There are long-standing debates about the role of non-profit and for-profit organizations in the health-care sector and changes in profit status (1, 2). For-profit organizations have a prominent presence in what is clearly a mixed ownership industry. Among non-government organizations, for-profits account for about 40% of U.S. hospitals (3) and 74% of U.S. nursing homes (4).

It is common to expect that all health-care providers are concerned with both financial performance and quality of care. Hirth (5) posits that non-profit nursing homes maximize quality subject to non-distribution constraints, placing concerns about quality ahead of concerns about profits. To test predictions on differences between non-profits and for-profit health-care organizations, most empirical studies have examined quality of care, charity care, and utilization of intensive care. Results are rather mixed. For example, Sloan et al. (6) find that non-profit hospitals have lower costs than non-profits, with no difference in the quality provided. Shen (7) finds that fewer adverse outcomes occur among acute myocardial infarction patients at non-profit hospitals. Norton and Staiger (8) find that ownership choices often interact with unobservable market-level characteristics. They also find that for-profit hospitals self-select into well-insured areas. Without controlling for such self-selection problems, estimates based on a direct comparison between non-profits and for-profits can be endogenous.

In this paper, we ask whether non-profit and for-profit nursing homes place different weights on financial and quality performance in deciding managers’ compensation. We suggest that managers might receive incentives that reflect the motives of their organizations. The idea is that if the non-profits truly pursue quality beyond its direct impact on financial performance, then quality of care should also be independently important in determining managers’ compensation. This idea is not completely novel, as compensation under different ownership structures has been previously explored. For example, Roomkin and Weisbrod (9) find that compensation for top executives is higher in for-profit hospitals than in non-profits. They also examine the compensation composition between base salaries and bonuses and find that bonuses are absolutely and relatively greater in the for-profits. Ballou and Weisbrod (10) examine chief executive officers (CEOs) compensation structure and find that religious non-profits pay significantly higher base salaries and that secular non-profits are more likely to provide bonuses and incentive plans. Preyra and Pink (11) find that CEOs of non-profit hospitals earn significantly lower but much more stable compensation than their counterparts at publicly traded companies. Kramer and Santerre (12) find that non-profit hospital CEOs’ pay is driven by the occupancy rate and admission of privately insured patients.

A limitation of many previous studies is their use of either single-year data (9, 10), or U.S. Internal Revenue Service Form 990 (13), or confine the scope of their study to only non-profit organizations (11, 14). Furthermore, prior studies have lacked information describing managers’ experience and educational background that may be useful in explaining differences in compensation that extend beyond financial and quality measures.

### Conceptual Framework and Hypotheses

It is widely recognized that managers are employed by the board of directors/trustees to act as the representative agents for the shareholders (in the for-profit case) or the donors and communities (in the non-profit case). Under the standard principal-agent model, the board/trustee is the principal who contracts with the agent (the manager) to make optimal use of resources and to maximize the share holders’ welfare (15). Because managers’ effort is not perfectly observable and monitoring managers’ behavior is often costly, managers may actually maximize their own benefits instead of shareholders’ welfare. It is not feasible to contract managers’ every effort, leading boards to seek a second-best alternative: compensation for performance. Performance-based compensation ties at least part of the managers’ compensation to outcomes that are observable to the board. In a profit-maximizing private firm, managers’ compensation is often tied to financial performance. This can take the form of performance-based cash bonuses or stock options that supplement base salaries. In a mixed ownership industry, the contract between the principal and the agent becomes more complicated, as non-profits can have motives as important, or more important, than profit-maximization (e.g., quality of care).

Because this paper uses nursing home data in the empirical analysis, we discuss hypotheses in the context of the nursing home industry. For-profits and non-profits are the two major organizational forms in the nursing home industry. We suggest that one key task of for-profit nursing home managers is to maximize profits. Thus, if the firms connect managers’ compensation to performance, managers’ compensation should be at least partially tied to nursing homes’ financial performance. The relationship between compensation and quality measures is less clear. Theoretically, quality performance only matters to for-profit nursing homes through its impact on financial performance. For example, good quality may attract patients who are willing to pay higher prices, and good reputation is an intangible asset that allows nursing homes to attract patients in the long term. It is possible that for-profit managers are indirectly rewarded for quality that improves profitability. Yet, managers in for-profits should be less likely rewarded for quality that is driven by non-financial motives.

We further suggest that non-profit organizations pursue some non-financial goals such as quality and community services. Under these circumstances, it is more difficult to tie optimal manager time and effort to each organizational goal. In the context of the nursing home industry, non-profit nursing homes are usually thought to maximize and balance both profits and quality. Although there are no shareholders in the non-profit nursing homes, earning profits is still an important goal, as profits supply financial resources needed to provide services and quality care. What sets non-profits apart from for-profits is that better quality itself can be a direct and independent goal, even if quality already exceeds a profit-maximizing level. In the non-profit nursing homes, both financial and non-financial motives make quality as an important objective.

**Hypothesis 1:** *Financial performance is expected have a stronger influence on for-profit managers’ compensation than that of non-profit managers. In turn, quality performance is expected to have a stronger influence on non-profit managers’ compensation than that of for-profit managers*.

### Owner-Managers

We further consider managers of for-profit nursing homes of being one of two types: those who have significant ownership (owner-managers) and those who do not (non-owner managers). A manager is considered as an owner-manager if the manager has at least a 5% ownership stake in the firm. Their incentives may differ from those of managers who do not have significant ownership (16). To develop a specific hypothesis, we borrow concepts from two different schools of thought: the optimal contracting approach and the managerial power approach. The optimal contracting model suggests that, because owner-managers can directly share a portion of the residual profits, the principal-agent problem may be milder (17, 18). Using a dataset of small corporations, Ang et al. (19) find that agency costs are inversely related to managers’ ownership share. Since there is less need to use pay-for-performance to mitigate the agency problem, one may expect that owner-managers’ compensation would be tied less to their performance (20).

The managerial power approach provides another hypothesis about owner-managers’ compensation (21, 22). Because owner-managers have more influence on corporate policies, they may be more likely to exercise their managerial power to collect private benefits, including their own compensation (23–27).

In addition, as compared to non-owner managers, owner-managers face higher risks from their equity stake. Owner-managers can also exercise their managerial power to raise compensation to reflect the investment uncertainty. The hypothesis about owner-managers can be synthesized as follows:

**Hypothesis 2:** *Owner-managers are expected to earn higher compensation than non-owner managers, with owner-managers’ compensation being less strongly tied to their performance*.

## Materials and Methods

We obtain a unique 8-year (2003–2010) dataset that provides detailed manager characteristics and compensation for all for-profit and non-profit nursing home in Ohio. The managers are the nursing home administrators who are the main decision makers and are responsible for the operation of the nursing home. The Ohio Department of Job and Family Services collects an annual cost report from every nursing home that receives state Medicaid reimbursement. Because this dataset contains information for both for-profit and non-profit nursing homes, we are able to compare directly the compensation and its relationship with financial and quality performance between for-profit and non-profit nursing homes.

**Figure 1:**
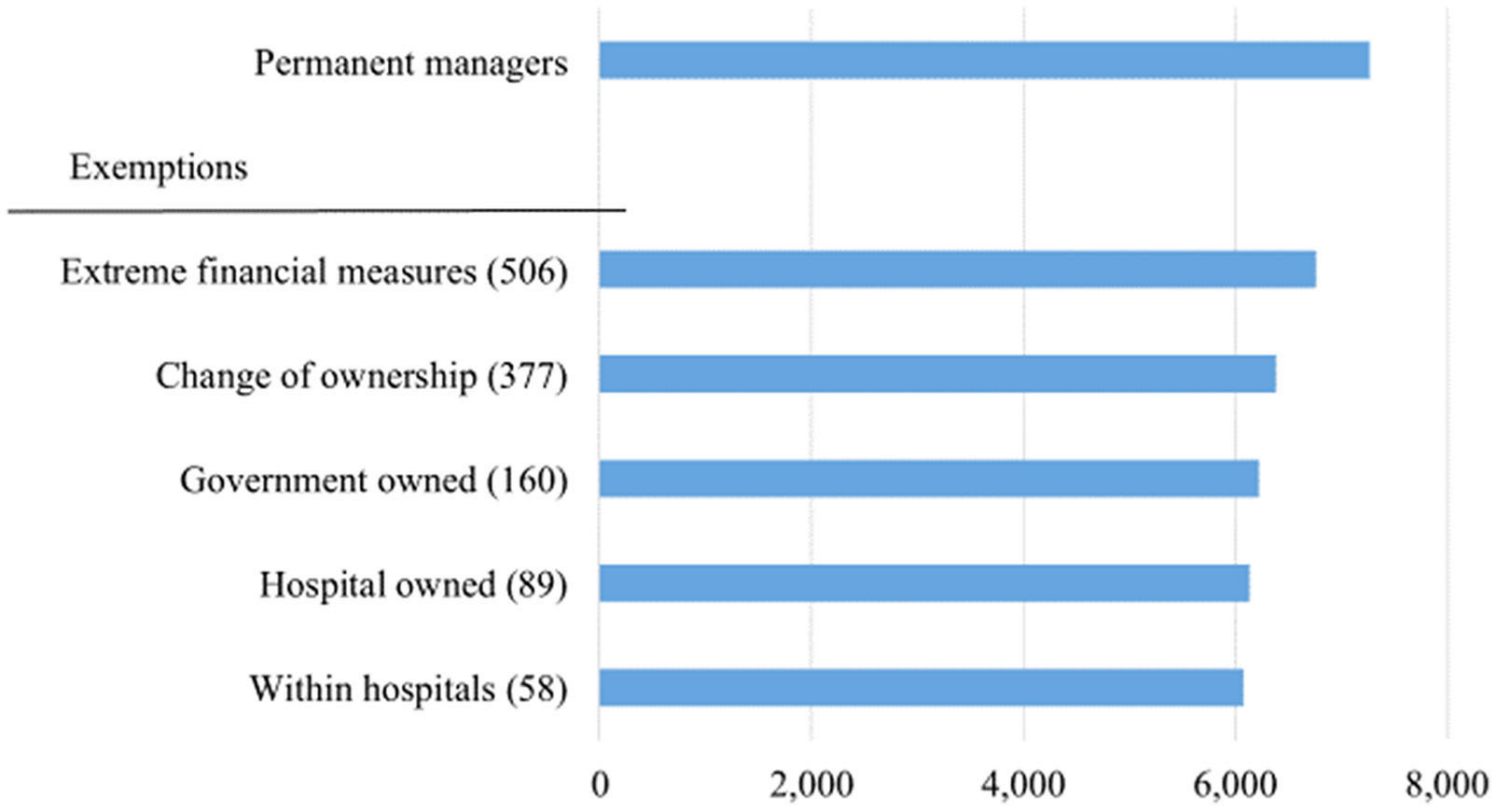
**Sample of managers and exemptions**.

These data include information on permanent managers (as opposed to interim managers), defined as managers who worked at least 200 days during each fiscal year under examination, resulting in 7,261 person-year observations. As depicted in Figure 1, several exemption criteria are employed to create a consistent set of comparisons. We exempted observations on extreme financial measures, defined as the top and bottom 1% values of compensation, assets, profit margins, and return on assets (ROA). We also exempted nursing homes changing their ownership, owned by a government entity, owned by a hospital and located within a hospital, resulting in an analytic sample with 6,071 observations.

The key dependent variable is annual compensation. Nominal values of compensation are adjusted to year 2003 dollars using the consumer price index. Other manager characteristics that may explain compensation include college education, years of health-care-related work experience, and ownership. College education is a binary variable associated with having a bachelor’s degree. The particular major or minor are not included, nor is there any consideration of graduate degrees. Years of work experience are reported in continuous numbers and capped at 10 years in the data. Owner management is defined as having more than a 5% equity stake in their nursing home’s equity.

Among many potential financial measures, we use both the profit margin and ROA as the primary proxies for financial performance. The profit margin is defined as net income divided by total revenues. The second financial measure, ROA, is defined as net income divided by total assets and has been widely used in prior research on managerial compensation (28, 29).

We use four common quality measures as proxies for managers’ performance on quality of care. These measures are the numbers and severity of health deficiencies identified by inspectors, prevalence of restraints, prevalence of pressure sores, and nurse hours per patient day. We extracted quality measures from the Center for Medicare and Medicaid Services’ Nursing Home Compare (NHC) website (https://www.medicare.gov/nursinghomecompare/). The website includes comprehensive quality measures for all Medicare-certified nursing homes. Data about inspection deficiency measures are available starting with 2001, and other quality measures are available from 2003 to 2010. Every 9–15 months, state health personnel inspect all nursing homes and report any deficiencies and their severity. We weight each deficiency according to its severity (from 1 to 12) and create a *Deficiency Score* variable that aggregates all severity-weighted deficiencies; the higher the deficiency score, the lower the quality. High prevalence of restraint use and pressure sores in general represents low quality.

We also use Online Survey and Certification and Reporting and NHC datasets to provide several important control variables, including the number of beds, chain affiliation, payer-mix, average number of activities of daily living (ADLs), for-profit market share, Herfindahl–Hirschman Index (HHI) as a measure of market concentration, median household income, and the population (in 1,000) above 65 years old per square mile.

### Empirical Specification

We use nursing home fixed-effect models in our analysis. The dependent variable is the managers’ annual compensation. The nursing home fixed-effect model can account for facility-level time-invariant variables that are not observable (e.g., corporate governance). The regression model is described as:

$$Y_{i,t}=\delta \cdot {\mathrm{Performance}}_{i,t-1}+\gamma \cdot {\mathrm{Firm}}_{i,t-1}+{\mathrm{Market}}_{m,t}+\Phi \cdot {\mathrm{Manager}}_{i,t}+\mu \cdot {\mathrm{Owner}}_{i,t}+H+T+\varepsilon_{i,t}$$

where *Y_i,t_* represents the level of managers’ annualized compensation. *Performance* includes the measures of the nursing home financial and quality performance. *Firm* is a vector of time-varying nursing home characteristics including size (number of beds), chain affiliation, continuing care retirement communities (CCRC), occupancy rate, and the payer mix among Medicaid, Medicare, and private payers. We lag the performance and nursing home-level variables by 1 year, because managers’ compensation may be based on previous performance. *Market* represents the market level characteristics, such as for-profit market shares, HHI, and demographic variables (the density of the elderly population) for market *m* in year *t*. We also include the count-level household median income to account for the difference in costs of livings, especially between rural and urban settings. We use county as the definition of the local market. *Manager* represents the manager-level characteristics, including a variable indicating whether the manager has a bachelor’s degree and a continuous variable of the managers’ years of work experience in a related field. *Owner* is one if the managers are also the owners. *H* represents a set of nursing home characteristics that are constant over time (e.g., for-profit status) and control for nursing home-fixed effects. *T* represents the year dummy variables that control for year-fixed effects and ε is the error term. Statistical analyses were performed using the STATA 14.1 (30).

## Results

Table 1 presents the means and SDs of the analytic sample. The CPI adjusted mean compensations are $70,278 and 67,096 for the for-profit and non-profit managers, respectively.

**Table 1:** **Summary statistics**.

	For-profit		Non-profit	
	Mean	SD	Mean	SD
**Compensation**				
Annual compensation (nominal)	78,105	30,838	74,220	27,177
Annual compensation (CPI adjusted)	70,278	27,318	67,096	23,959
**Performance measures**				
**Financial measures (%)**				
Return on assets	13.75	44.77	0.58	19.15
Profit margin	3.41	8.70	1.79	11.30
**Quality measures (%)**				
Deficiency score	22.19	20.54	17.42	17.61
Prevalence of restraint	5.39	5.99	3.82	5.14
Prevalence of pressure sores	8.96	7.83	8.59	6.86
Nurse h/day	3.62	0.91	4.10	1.22
**Firm characteristics**				
Private-pay share (*t* − 1)	0.21	0.12	0.30	0.16
Medicaid-pay share (*t* − 1)	0.67	0.15	0.58	0.18
Average activities of daily living (ADLs) (*t* − 1)	5.32	0.80	5.37	0.78
Chain	0.64	0.48	0.45	0.50
Occupancy rate (*t* − 1)	86.74	12.06	91.06	11.06
Number of beds (*t* − 1)	98.71	43.24	103.92	54.49
Continuing care retirement communities	0.02	0.15	0.15	0.36
**Market characteristics**				
For-profit market share	0.89	0.19	0.41	0.33
000’ 65+ per square mile	0.12	0.13	0.14	0.13
Herfindahl–Hirschman index	0.40	0.30	0.40	0.28
Log (median income)	10.69	0.15	10.71	0.15
**Manager characteristics**				
Bachelor degree	0.90	0.30	0.93	0.26
Years of work experience	8.76	2.39	9.23	1.87
Owner	0.18	0.38	NA	NA
**Number of observations**	5,027		1,044	

*Private-pay share, Medicaid-pay share, average ADLs, occupancy rate, and number of beds are 1 year lagged values (*t* − 1) and therefore have fewer observations*.

The average profit margins are 3.41 and 1.79% and the average ROAs are 13.75 and 0.58% for the for-profit and non-profit nursing homes, respectively. While profit margin and ROA are potentially closely related, the correlation coefficient between these two financial measures is only 0.118, permitting the inclusion of both measures in multivariate analyses.

On average, for-profit nursing homes have lower quality than their non-profit counterparts. In for-profit nursing homes, about 5.39% residents are physically restrained, 8.96% have pressure sores, and total nurses hours average 3.62/day. In non-profit nursing homes, only 3.82% of the residents are physically restrained, 8.59% of the residents have pressure sores, and total nurse hours average 4.10/patient day. As with the financial measures, the quality measures are not highly correlated, and all measures are included in the regressions.

Many of the firm-level and market-level control variables exhibit similar values between the for-profit and non-profit nursing homes. Medicaid market share, ADLs, occupancy rates, number of beds, population (in 1,000) above 65 years old per square mile and HHI are similar between ownership types. For-profit nursing homes exhibit a lower percentage of private pay patients, are more likely to be part of a chain and less likely to be part of a CCRC. For-profit nursing homes are also more likely to be an area with a larger concentration of for-profit nursing homes.

Over 90% of managers hold a college degree with non-profit managers being slightly likely than for-profit managers to hold a college degree. Non-profit managers also have more health-care-related work experience. Nearly one-fifth of the for-profit managers have more than a 5% equity stake in their nursing homes equity and are considered the owners of the nursing facilities.

The regression results in Table 2 show that for-profit managers’ compensation is positively associated with better financial performance, but is not related to quality. On the other hand, we find no consistent relationship between non-profit managers’ compensation and either financial or quality performance.

**Table 2:** **NH fixed-effect: performance and managers’ compensation ($)**.

	All	For-profit	Non-profit

	(1)	(2)	(3)
**Performance**			
Profit margin (*t* − 1)	2.91**	3.87***	−33.07***
	[1.359]	[0.659]	[12.629]
ROA (*t* − 1)	2.96**	2.90**	21.04
	[1.379]	[1.394]	[16.781]
Restraint (*t* − 1)	70.66	92.76	−91.1
	[58.982]	[65.309]	[134.371]
Pressure sores (*t* − 1)	14.53	6	41.47
	[50.416]	[57.067]	[88.511]
Deficiencies (*t* − 1)	−1.26	0.2	−7.37
	[14.117]	[15.457]	[28.171]
Nurse h/day (*t* − 1)	−193.93	−404.4	167.21
	[309.421]	[414.884]	[355.446]
**Firm characteristics**			
Chain (*t* − 1)	116.56	635.93	−3,395.72
	[1,138.520]	[1,252.867]	[2,249.454]
Occupancy rate (*t* − 1)	95.73***	110.90***	−68.17
	[34.050]	[37.011]	[76.668]
Number of beds (*t* − 1)	98.25**	88.18**	141.88*
	[40.089]	[43.674]	[78.543]
Private-pay share (*t* − 1)	−2,688.23	−2,359.93	3,381.55
	[4,653.277]	[4,756.397]	[13,018.511]
Medicaid-pay share (*t* − 1)	−5,175.33	−4,357.44	−3,825.85
	[4,518.687]	[4,707.430]	[11,770.660]
Avg. # of activities of daily living (*t* − 1)	−183.49 [456.755]	−329.4 [480.068]	1,057.54 [1,344.722]
**Manager characteristics**			
Owner	17,295.24***	17,369.93***	
	[3,253.059]	[3,223.253]	
Bachelor degree	−3,391.87	−3,778.66	−394.31
	[2,401.295]	[2,848.117]	[2,179.143]
Experience (years)	1,447.64***	1,473.50***	1,208.66***
	[211.830]	[232.309]	[320.088]
*R*-squared	0.29	0.29	0.35
Observation	6,022	5,020	1,002

(1) ***, **, and * represent significance at 1, 5, and 10% levels, respectively.

*(2) We use robust SE for nursing home fixed-effect models*.

*(3) All regressions control for the year-fixed effects, county level income, percentage of population above 65 years old, Herfindahl–Hirschman Index, and whether or not the facility is part of the continuing care retirement communities*.

Among the firm-level variables, occupancy rate, number of beds, and payer mix are the most influential variables. Consistent with prior studies [e.g., Ref. (10)], firm size is an important factor in determining the compensation. The different mix among Medicaid, Medicare, or private payers implies that the nursing homes have different patient-mix and therefore require managers to have differential skills and abilities. The coefficients of the private-pay and Medicaid-pay shares are both negative and significant (at least among for-profits), suggesting managers’ compensation is positively correlated with Medicare-pay shares. Particularly, Medicare covers post-acute care for 100 days, and residents engaging in this care often require more complicated services and special rehabilitations. The results suggest that managers of nursing homes with higher Medicare revenue share receive significantly higher compensation.

All else equal, the results in Table 2 indicate that owner-managers, on average, earn about $17,295 more than their non-owner peers. Having a college degree is not associated with a statistically significant pay differential. Experience matters with a 1 SD more of work experience (2.4 years) being associated with about 4.9% ($3,474) higher compensation.

We further split the for-profit nursing homes into two samples: administered by owner-managers and non-owner managers. We perform a sub-analysis to examine whether the relationship between compensation and performance differs between owner-managers and non-owner managers. The results are presented in Table 3. We find that the compensation of both owner-managers and non-owner managers are not related to quality performance. In terms of financial performance, the compensation of non-owner managers is significantly and consistently correlated with profit margin and ROA but, on the other hand, the compensation of owner-managers does not have consistent and significant relationship with profit margin and ROA. Overall, our results provide suggestive evidence that owner-managers earn higher compensation and it is less tied to both financial and quality performances.

**Table 3:** **NH fixed-effect: performance and managers’ compensation ($) by owner and non-owner manager**.

	Owner-manager	Non-owner manager

	(1)	(2)
**Performance**		
Profit margin (*t* − 1)	−147.99*	2.80***
	[80.779]	[1.014]
ROA (*t* − 1)	28.24	4.50***
	[22.653]	[1.661]
Restraint (*t* − 1)	−54.5	94.76
	[315.173]	[81.552]
Pressure sores (*t* − 1)	37.49	−5.15
	[252.709]	[70.770]
Deficiencies (*t* − 1)	−24.28	−0.31
	[63.417]	[20.135]
Nurse h/day (*t* − 1)	328.24	−41.21
	[1,802.517]	[536.768]
**Firm characteristics**		
Chain (*t* − 1)	−3,628.71	512.09
	[4,822.441]	[1,471.581]
Occupancy rate (*t* − 1)	360.61**	76.1
	[149.671]	[46.921]
Number of beds (*t* − 1)	324.45**	120.04**
	[135.165]	[54.090]
Private-pay share (*t* − 1)	−9,943.21	−10,675.86*
	[22,441.546]	[6,454.507]
Medicaid-pay share (*t* − 1)	−13,972.39	−8,558.53
	[18,823.761]	[6,469.306]
Avg. # of activities of daily living (*t* − 1)	−1,911.47 [2,492.039]	−1,165.66* [629.276]
**Manager characteristics**		
Bachelor degree	−15,014.35	−4,648.48**
	[12,435.582]	[1,993.941]
Experience (years)	4,238.11***	1,462.97***
	[1,533.276]	[230.949]
*R*-squared	0.23	0.37
Observation	868	4,156

*The sample is limited to for-profit nursing homes*.

(1) ***, **, and * represent significance at 1, 5, and 10% levels, respectively.

*(2) The SE is clustered at the county level*.

*(3) All regressions control for the year-fixed effects, county level income, Herfindahl–Hirschman Index, for-profit market share, percentage of population above 65 years old, and whether or not the facility is part of the continuing care retirement communities*.

## Discussion

Overall, we find that in the nursing home industry, managers’ compensation reflects common financial measures among for-profit homes, but the magnitude of the effect is relatively small. For a for-profit manager, a 1 SD increase in profit margin is associated with $33.67 higher compensation and a 1 SD increase in ROA is associated with $129.83 higher compensation. The results are consistent with the literature (11, 31) suggesting that firms are more reluctant to use performance-based incentives when the managers are contracted over several tasks, especially when some of them are difficult to measure. In addition, it is possible that resident satisfaction and other dimensions of quality are used in evaluating managers’ performance, but they are not included in our analysis.

From the perspective of both organizational governance and regulatory overseers, the results of this study are both affirming and potentially concerning. Appropriate compensation of senior managers is a key function of governance. Observing that compensation is closely related to years of experience and the size of the enterprise is just as one would expect in a well-functioning market for managers. The observation that education is either not related to compensation or negatively associated with compensation is somewhat surprising.

The first part of hypothesis 1 is confirmed that financial performance has a stronger influence on for-profit managers’ compensation than that of non-profit managers. The second part is not confirmed, as quality performance does not appear to have a stronger influence on non-profit managers’ compensation than on for-profit managers’ compensation. While some of the coefficients are in the hypothesized direction, all estimates are far from statistically significant and do not provide strong support for quality as an independent determinant of manager compensation.

The lack of significant relationships between non-profit managers’ compensation and both financial and quality performance suggests that non-profit nursing homes may not reward or penalize their managers based on performance. This is a potentially troublesome finding. Both organizationally and socially, provision of high quality nursing home services is very important. These data do not suggest that quality levels are so high that there is a ceiling effect that does not permit observation of the association between quality and compensation – the association simply does not present itself in these data. More refined attempts to establish this association are worthy of future attention.

Hypothesis 2 is confirmed that owner-managers earn higher compensation than non-owner managers and that owner-managers’ compensation tied less strongly to their performance. Compensation of non-owner managers is significantly and consistently correlated with profit margin and ROA, but the compensation of owner-managers does not have consistent and significant relationship with profit margin and ROA.

With regard to the higher compensation paid to owner-managers, the observation may simply be an accounting choice issue. That is to say, firms can choose to distribute earnings through higher salaries of managers or as dividends/profit distributions. Setting aside tax implications, owners may be indifferent between these options and, on average, provide earnings through managers’ salaries. Given this choice of distributing some profits through salaries, reported profits among owner-managed firms may be lower than would otherwise be the case.

Including profits as a determinant of compensation is common practice among many firms. Among non-profits, measures of budget adherence, rather than profits, is quite common. Again, the finding of a negative relationship between compensation and profit margin for owner managers may be explained by the choice of profit distribution.

### Limitations

There are several limitations to the conclusions of this study. First, we do not have data on managers’ tenure, turnover, and other personal characteristics that could potentially be significant, and their absence can cause omitted variable biases. Compensation might be positively correlated with tenure in the same firm. If non-profit managers were to stay in the same organization for a longer period than for-profit managers, the compensation comparison between for-profit and non-profit could be biased. It is possible that performance is reflected in managers’ turnover but not in their compensation. Furthermore, there are a host of other personal characteristics, such as age and gender that may be related to compensation but are not captured in available data.

Second, the compensation information does not separate the base salaries and stock or other bonus. For privately held nursing homes, without the market value of the stock bonus, the actual compensation that includes stock bonus can be potentially higher than reflected in the data. It is also possible that performance is tied to the composition of compensation and not the overall level of the compensation. Although Cole and Mehran (32) point out that only very few privately held small firm issue stock options, there are nursing homes belonging to national chains and their managers might be more likely to receive stock bonus. Unfortunately, such detailed chain information is not available in our data. While we include the chain affiliation as an independent variable, we are not able to identify the large and publicly listed chains. Managers at the publicly listed and privately held chains may receive different compensation.

Third, while the current reports are assumed to be consistent among for-profit and non-profit organizations, ownership type may influence reporting practices. There is evidence that for-profit hospitals report some financial data more aggressively than non-profit hospitals (33), and the same may be true for this sample of nursing homes.

Finally, because our analysis relies on Ohio nursing homes only, the results may not be generalized to other states or other nations, where may have very different policy regulations and market structures.

## Conclusion

The primary goal of this paper is to use a novel dataset of managers’ compensation to empirically examine whether for-profit and non-profit organizations place different emphasis on financial and quality motives. We find that better financial performance is associated with slightly higher compensation among for-profit managers. Our results provide supportive evidence that for-profit managers are contracted and rewarded differentially from those working for the non-profits. The lack of significant relationship between compensation and quality metrics at both for-profit and non-profit nursing homes is potentially troublesome. However, the results should be interpreted with caution due to data limitations. Furthermore, the evaluation and monitoring of quality performance at the facility level may be too costly to the board because quality is multi-dimensional, many health outcomes often depend on the health status of patients when admitted, and the documentation and evaluation of every quality metric might just not always be feasible. These factors and others may prevent managers’ compensation from reflecting short-term quality performance.

## Author Contributions

The authors all contributed to the design, analysis, and writing of the manuscript.

## Conflict of Interest Statement

The authors declare that the research was conducted in the absence of any commercial or financial relationships that could be construed as a potential conflict of interest.
